# Numeric and morphological studies of the African lion (*Panthera leo leo*) pectoral limb

**DOI:** 10.1186/s12917-022-03488-x

**Published:** 2022-11-05

**Authors:** Kenechukwu Tobechukwu Onwuama, Esther Solomon Kigir, Alhaji Zubair Jaji, Suleiman Olawoye Salami

**Affiliations:** Department of Veterinary Anatomy, Faculty of Veterinary Medicine, University of Ilorin, Ilorin, Nigeria

**Keywords:** Anatomy, Bone, Metacarpal, Humerus, Phalanges

## Abstract

**Background:**

The Importance of the appendicular skeleton in the conformation, shape and physiology of wild animals especially carnivores for adaptation and survival cannot be overemphasized, as limited and obsolete information on the bones of the forelimb necessitated this study. Matured adult African lions (Male and female) that weighed 173 kg and 112 kg were obtained at different times after post mortem examinations of their carcasses. Bone preparation was achieved via cold water maceration after proper skin and muscle removal to a barest minimum.

**Results:**

The acromion process of the scapula consisted of a ventral hamate and caudal suprahamate processes. The Clavicle was absent in this species. The musculospiral groove of the humerus was more or less absent while its supracondyloid foramen and crest were positioned above the medial condyle and lateral condyle respectively. The radio-ulna presented a twisted appearance with the radius slightly curved thereby creating an extensive interosseous space that spanned its entire length. The seven (7) carpal bones were uniquely arranged in two rows while the 5 metacarpals anchored 5 digits with 3 phalanges except the first with 2 phalanges each. Two sesamoid bones were located on the ventral surface of each Metacarpophalangeal joint. None was seen on its dorsal surface. The 3rd phalanx had a unique appearance with a crescent plate projecting from the ventral cavity. The average total number of bones constituting the pectoral limb was 80.

**Conclusion:**

Numerical information and detailed anatomical features of the pectoral limb bones of the African lion (*Panthera leo leo*) have added some valuable literature to science. This further serves as a baseline data for future scientific exposition on this species.

## Background

The rapid decline of the African lion (*Panthera leo leo*), a member of the family Felidae, order Carnivora and suborder Feliformia [1] due to human domination and landscape fragmentation has raised conservation concerns [2]. The increasing probability of gradual extinction of this species [3] reduces its economic significance which stems from its recognition in human culture [4]. These concerns have led to archive of various parts of this species in natural history museums, exhibition centers and tertiary institutions around the world [5].

Being therefore one of the largest carnivore and best studied mammalian species in Africa [6], anatomical and morphological investigations have been conducted on the heart [7], reproductive organs [8], hyoid apparatus and pharynx [9], axial and hindlimb skeleton [10, 11] with the aim of proper documentation for knowledge acquisition and reference. Also, the African Lion’s characteristics, significance and behaviour further increases its interests in scientific investigations. To therefore further investigate adaptive features for prey hunting, carriage, movement and survival, the appendicular skeleton which is the framework by which other anatomical structures are built needed to be documented. To this end, this study on the pectoral limb was conducted to establish normal morphological features and numerical information on bones constituting this region of the African Lion skeleton. It serves as a base line information for archeological researches, academic teaching, wildlife forensic studies and identification when compared with other carnivores of same suborder or family.

## Materials and methods

Two adult (Male and female) African Lions (*Panthera leo leo*) weighing 173 kg and 112 kg with estimated ages of 6 and 4 years respectively were obtained as post-mortem carcasses at different periods from the Department of Veterinary Pathology, University of Ilorin, Nigeria. Bone processing was achieved through cold water maceration [12] which first entailed careful dissection to remove skin and muscles using surgical blade; thereby harvesting bones with minimal soft tissue attachment. These were then kept in a bucket of cold water enough to submerge the bones at room temperature. The bucket was then covered and placed under the sun throughout this process with regular water change. After some days, drainage of the water was done to recover and sundry bones of the pectoral limb. Counting of bones and detailed description of morphological features was performed following the standard nomenclature from Nomina Anatomica Veterinaria [13]. prior to Photographic documentation via a digital camera (COOLPIX NIXON 20 megapixel). Articulation via glue of some regions were also performed for proper understanding.

## Results

The Pectoral limb comprised bones of the Shoulder (Scapula), Arm (Humerus), Fore arm (Ulna and Radius), and Manus (carpals, metacarpals and phalanges). The clavicle was absent in this species. The bones contained morphological features that resembled and differed from other domestic animals studied. The total number of pectoral limb bones in this species was investigated to be 80 as represented in Table 1.

**Table 1:** Number of bones of the right pectoral limb of the Lion (*Panthera leo*)

Bones	Number
Scapula	1
Humerus	1
Ulna	1
Radius	1
Carpals	7
Metacarpals	5
First phalanx	5
Second phalanx	4
Third phalanx	5
Sessamoid bones	10
**Total:**	**40**

**Fig. 1:**
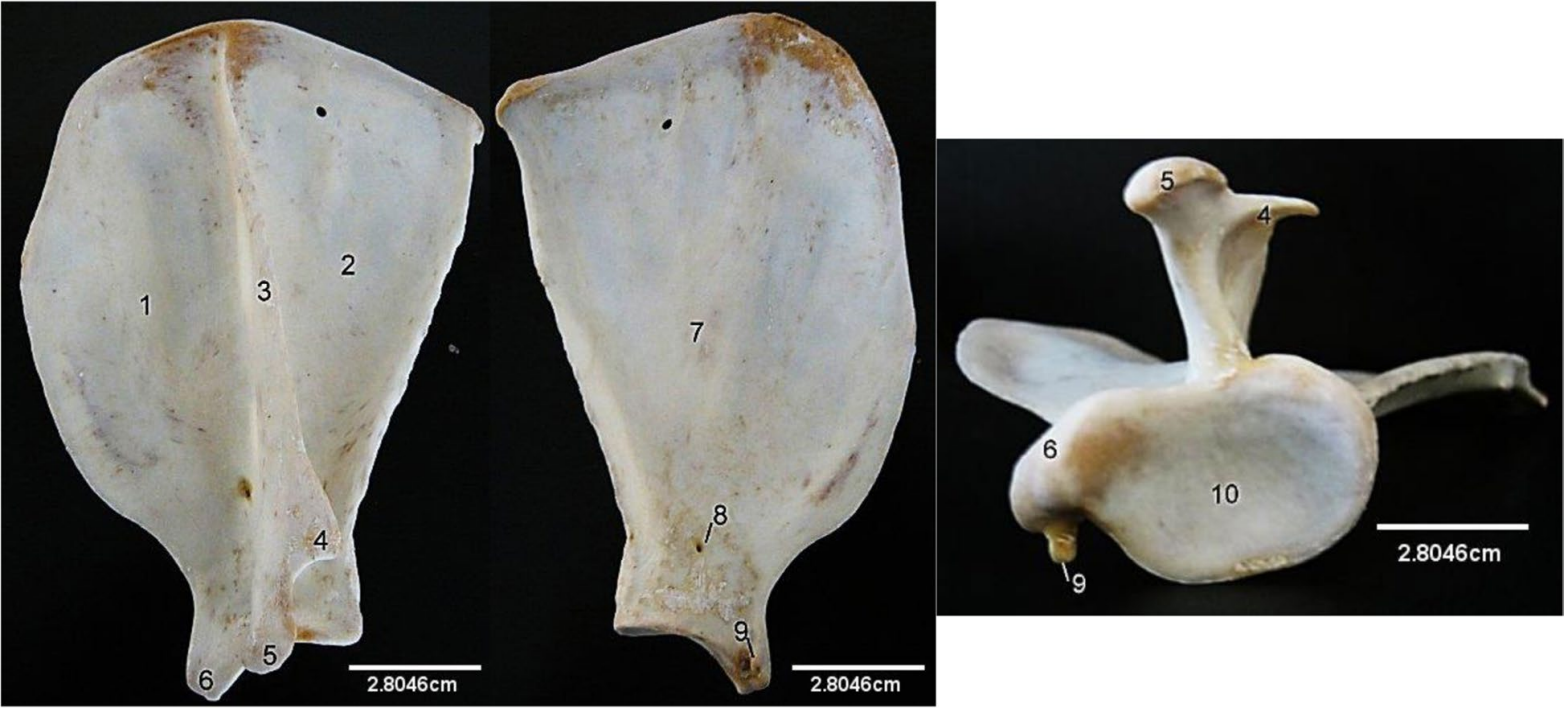
African Lion Scapula, Lateral (**A**), medial (**B**) and ventral (**C**) views. 1, Supraspinous fossa; 2, Infraspinous fossa; 3, Spine; 4, Suprahamate process; 5, Hamate process; 6, Post-glenoid tubercle; 7, Subscapular fossa; 8, Nutrient foramen; 9, Coracoid process; 10, Glenoid cavity

The Scapula (Fig. 1) presented a flat somewhat D-shaped bone. The caudal border was straight in outline while the cranial border was straight proximally, convex at its middle and concave distally. The dorsal border which lacked the scapular cartilage also assumed a convex outline. The lateral surface presented a prominent bony structure, the scapular spine which originated from the dorsal border and ended with the formation of acromion process. This consisted of a downward hamate process and a caudal suprahamate process. The scapular spine divided the lateral surface into two almost equal parts; the cranial supraspinous fossa and caudal infraspinous fossa. On the ventral angle and border (Fig. 1), the bone presented the neck, glenoid cavity, supraglenoid tubercle laterally and coracoid process medially. The medial surface of the bone presented a shallow thin fossa, the subscapular fossa at the centre and a tiny ridge close to the cranial border. A nutrient foramen was noticed at the ventral angle medially.

**Fig. 2:**
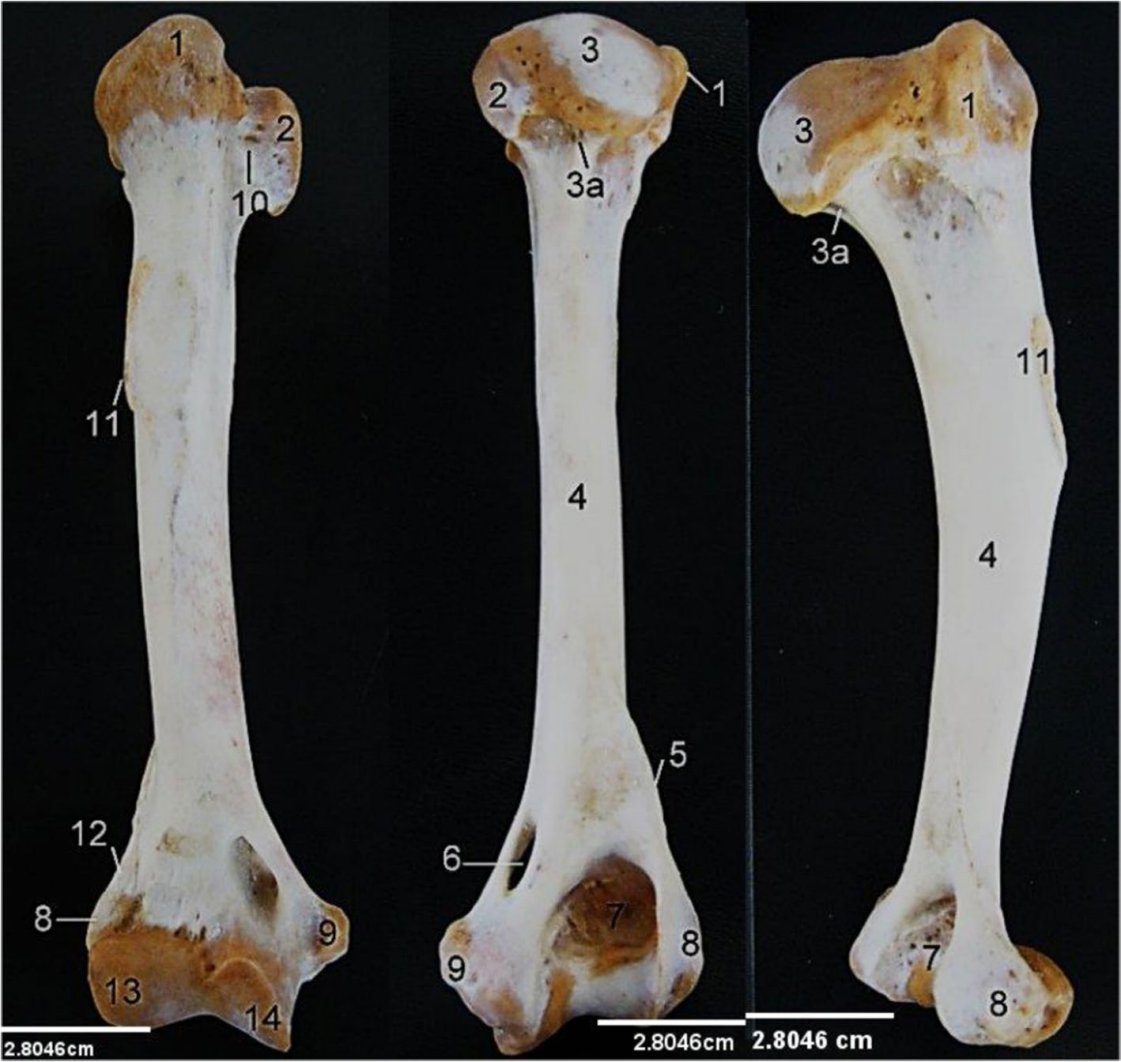
Lion Humerus, Cranial, caudal and lateral views. 1, Greater tubercle; 2, Lesser tubercle; 3, Head; 3a, Neck; 4, Body; 5, 12, Supracondyloid crest; 6, Medial supracondyloid foramen; 7, Olecranon fossa; 8, Lateral epicondyle; 9, Medial epicondyle; 10, Intertuberal groove; 11, Deltoid tuberosity; 13, Lateral condyle; 14, Medial condyle

The Humerus (Fig. 2) presented a long bone with two extremities (proximal and distal) and a shaft or body. The proximal extremity presented a caudally placed large convex head with a well-defined neck, lesser tubercle cranio-medially and greater tubercle caudo-laterally. An intertuberal groove separated the greater tubercle from the lesser tubercle medially. The greater tubercle was made up of two parts (cranial and caudal). The body presented a convex cranial and concave caudal surface. Towards the middle of the cranial surface, was a crest-like deltoid tuberosity that continues up to the lesser tubercle on the lateral surface. On its medial surface and adjacent to the deltoid tuberosity was the less prominent teres tubercle. The musculospiral groove was almost non-existent. On its distal third, it presented a distinct supracondyloid crest (ending at the lateral epicondyle) and supracondyloid foramen above the medial condyle. The distal extremity presented cranially the medial and lateral condyles, lateral and medial epicondyles and Olecranon fossa caudally.

**Fig. 3:**
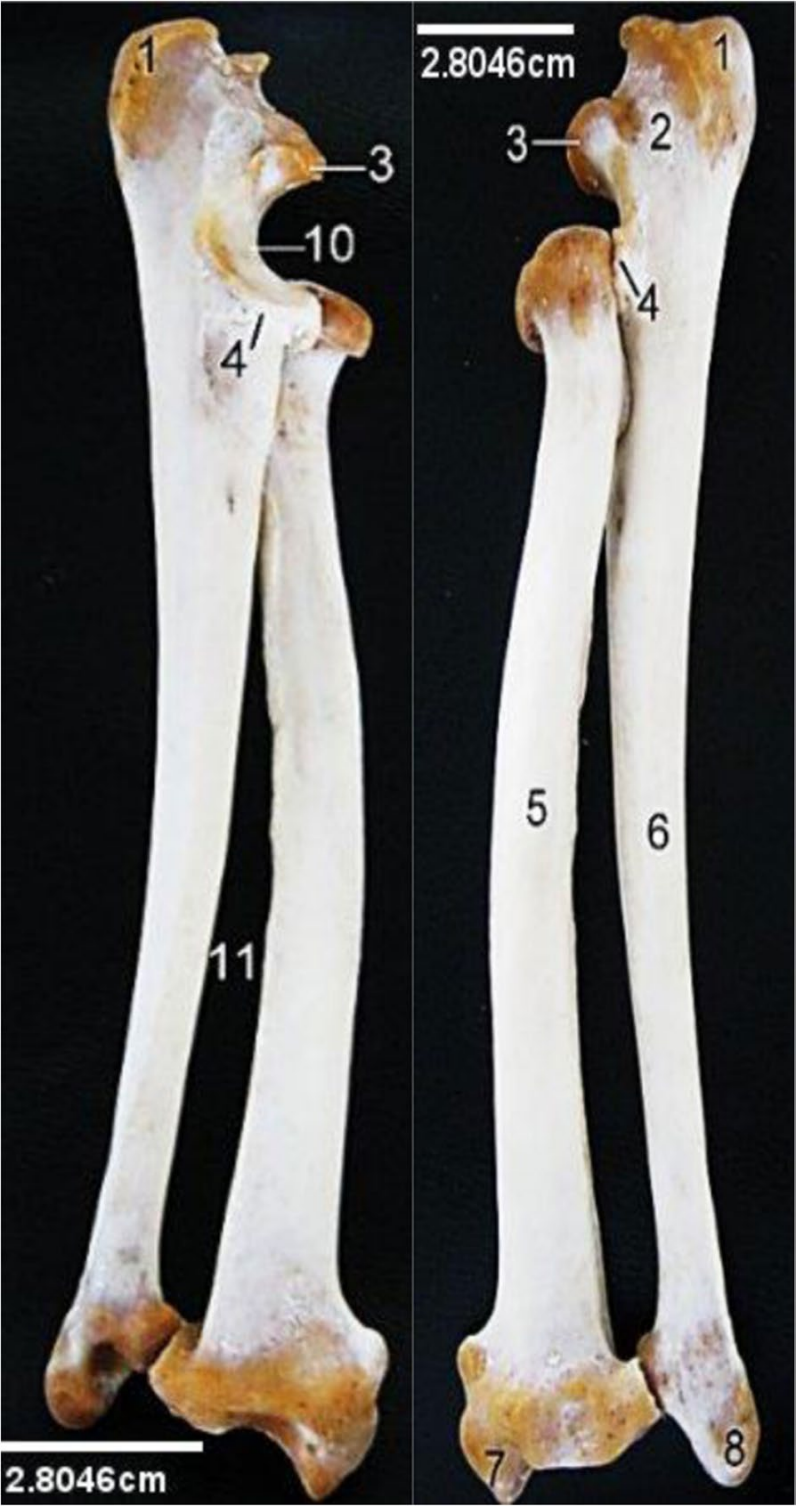
Lion Ulna and Radius (Medial and lateral views). 1, Olecranon tuber; 2, Olecranon; 3, Anconeal process; 4, Coronoid process; 5, Body of radius; 6, Body of ulna; 7, Styloid process of radius; 8, Styloid process of ulna; 10, Sigmoid notch; 11, Interosseus space

The Ulna and Radius (Fig. 3) were fused proximally and distally creating an extensive interosseus space and a rather twisting appearance. Both bones presented proximal and distal extremity attached to a long shaft. The proximal extremity of the ulna bone presented a small Olecranon ending in a beak shaped anconeal process cranially above the sigmoid notch which was formed entirely by the ulna while the capsular fossa of the radius attached laterally to it. Coronoid process projected lateral and medial from the notch. The radius presented proximally, radial articular facet (capsular fossa), radial tuberosity cranially, lateral and medial eminence for muscular attachment. Distally, the ulna and radius presented lateral and medial styloid processes respectively. The bodies of the bones appeared similar in size however the ulna had a larger proximal extremity while radius had a larger distal extremity.

**Fig. 4:**
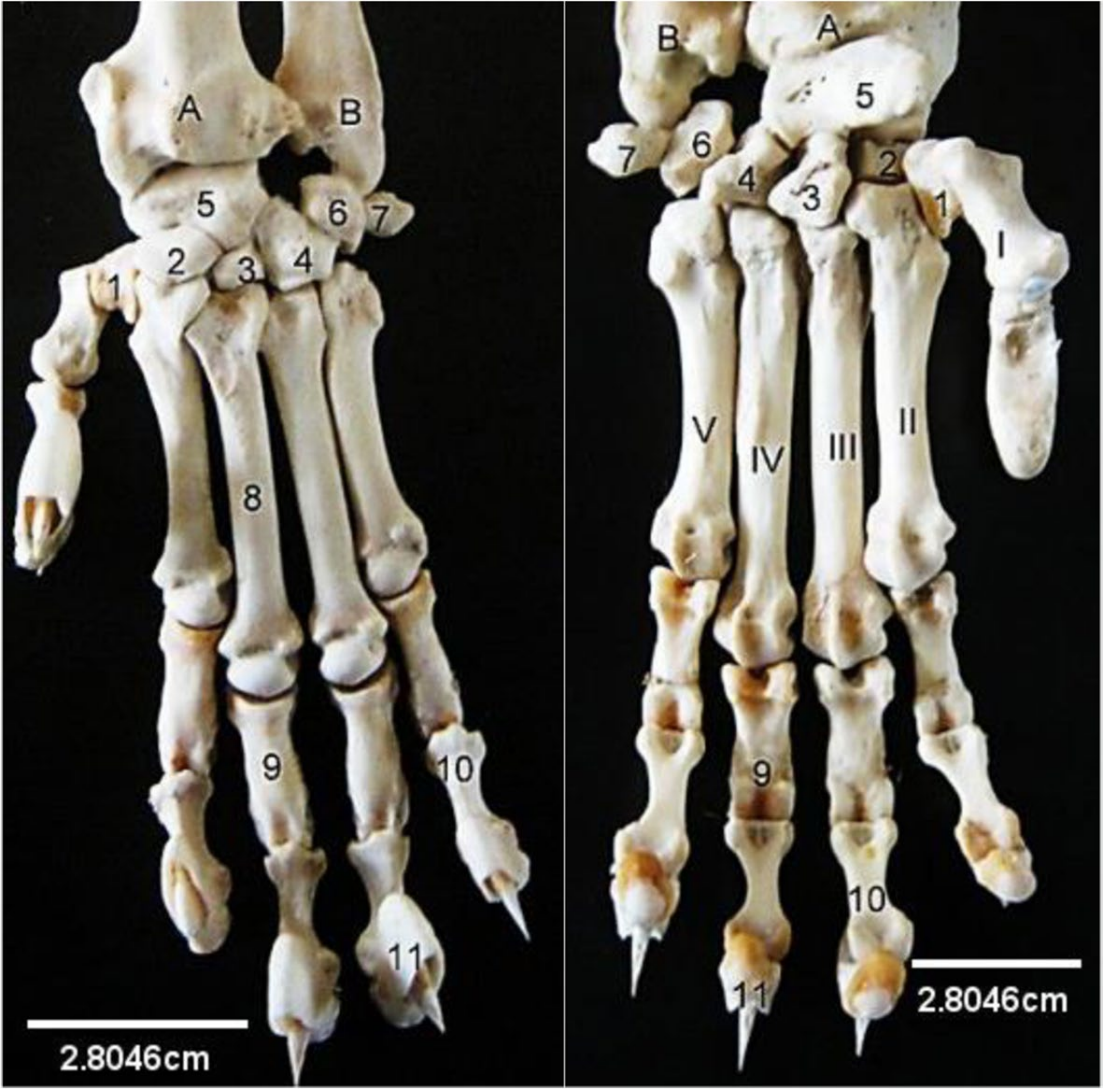
Lion Manus (Dorsal and Palmer view). A, Radius; B, Ulna; I-V, First to fifth metacarpals; 1, First carpal; 2, Second carpal; 3, Third carpal; 4, Fourth carpal; 5, Fused radial and intermediate carpal; 6, Ulna carpal; 7, Accessory carpal; 8, Third metacarpal; 9, Proximal phalanx; 10, Middle phalanx; 11, Distal phalanx

The Carpus (Fig. 4) presented seven bones arranged in two rows. The proximal row was made up of fused radial and intermediate carpal, ulna carpal and accessory carpal bone. The distal row comprised of first carpal bone, second carpal bone, third carpal bone and fourth carpal bone. The proximal row articulated with distal end of radius and ulna in the antebrachiocarpal joint while the distal row articulated with the metacarpal to form carpometacarpal joint. The fused radial and intermediate carpal bone was the largest among the carpal bones and it articulated with the distal part of the radius. The first carpal bone appeared small and attached to the first metacarpal bone at its most proximal aspect. The second carpal bone was larger than the first carpal bone and it was attached to the intermediate carpal bone proximally and distally with the first metacarpal bone. Third carpal bone was located on the proximal part of the second metacarpal bone and articulated with some part of the intermediate carpal bone, fourth carpal bone articulated with third carpal bone at its lateral side and with intermediate at its most proximal part and lastly with the ulna carpal bone dorsomedially. Ulna carpal bone attached to the fourth carpal bone at its ventral surface, accessory carpal bone which was the smallest amongst the carpal bone articulated with the ulna carpal bone and ulna bone.

**Fig. 5:**
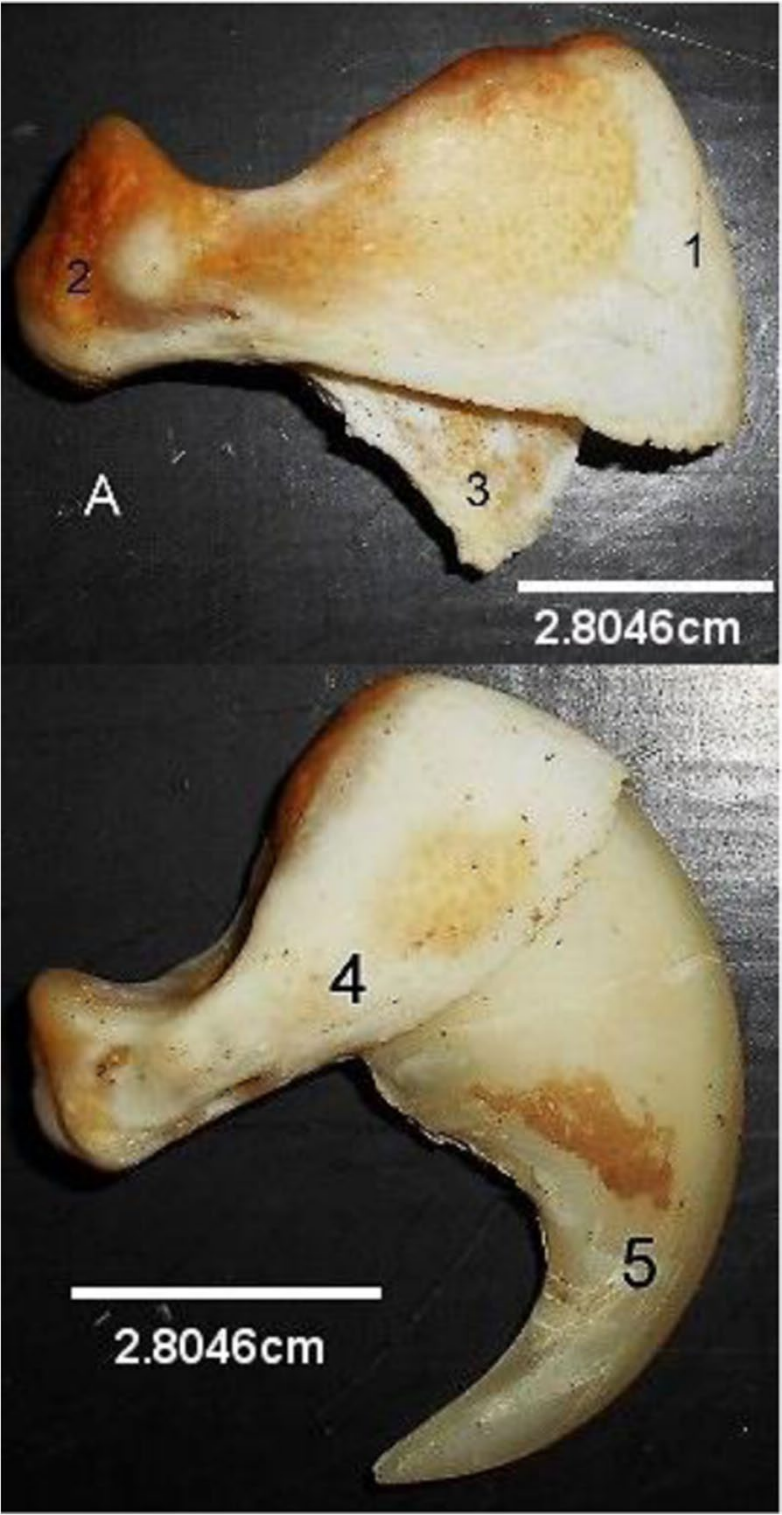
Lion Third Phalanx without claw (**A**) and with claw. (Lateral views). 1, Cranial Extremity; 2, Caudal tubercle; 3, Ventral plate, 4, Third phalanx; 5, Claw

Five (5) Metacarpal (Fig. 4) bones were present that anchored 5 digits. The first metacarpal was the smallest while the third and fourth metacarpal were the longest. The first digit comprised two phalanges while the rest (second to fifth) digits comprised three phalanges each. The third Phalanx (Fig. 5) had a characteristic appearance of a capsized boat with a wide convex cranial extremity and a caudal tubercle. The dorsal border was convex cranially before presenting an articular depression for the second phalanx articulation. A flat crescent plate (covered by a comma shaped claw in the live animal) projected ventrally from the open ventral border. Two sesamoid bones were located on the ventral surface of each Metacarpophalangeal joint. None was seen on its dorsal surface.

## Discussion

This study presented unique morphological features of the African Lion’s pectoral limb that appeared similar and different from other feline and canine species. The Scapula’s presentation of a straight caudal border, convex cranial, convex dorsal border and two surfaces with the lateral divided into two equal halves by the scapula spine was similar to that of the dog [14] and cat [15]. However, the division of the acromion process into hamate and suprahamate processes was only noticed in the cat, tiger and leopard [16, 17] suggesting that it may be a specific feature of feline species which helps to properly anchor the muscles attached to these processes and support the scapulohumeral joint which is actively involved during prey capturing. The acromion and hamate processes point cranio-distally unlike in the domestic cat [15] where it points directly distally. Kirberger [18] observed a bony protuberance on the proximal part of the caudal border which was absent in the specimen used. The absence of this feature corroborates the ages of the specimen used as such features can only be seen in old adults above 8 years. The caudal border in this species was roughened for possible attachment of the long head of triceps muscle. The scapula cartilage which was not observed in this specimen was documented by Kirberger [18] to be found above the margo dorsalis as a mineralized band in fewer older lions. The coracoid process observed on the medial aspect of the glenoid angle was not distinct when examined radiographically [18]. This further buttresses the importance of revealing the actual bones to identify specific features.

The musculospiral groove which is usually present in the humerus of the horse, pig and ruminants [19] was more or less absent in this species. This finding was similar to the presentation reported in the Tiger [20], cat [21] and dog [22]. However, the position of the supracondyloid foramen above the medial condyle and the supracondylar crest laterally on the distal extremity was similar only to the cat’s humerus [15]. The lateral supracondylar crest was absent while the supracondyloid foramen was located in the olecranon fossa of the dog [23].

The features of the radio-ulna bone which included the olecranon, anconeal process, coronoid process, medial and lateral styloid processes were all similar to those of the cat and dog [24]. However, uniqueness was found in their twisted appearance and slightly curved radius, which created an extensive interosseous space that spanned its entire length. The closure of the physis observed in this specimen further supports the finding by Kirberger [18] that all physis close at 54 months of age.

The presentations of the seven (7) carpal bones uniquely arranged in two rows and five (5) metacarpals with the first anchoring 2 phalanges while the remaining anchored 3 phalanges were similar to those of the cat and dog [19]. However, the third Phalanx’s characteristic boat-like appearance with a sharp pointed ventro-cranial end was similar only to that of the cat [15], suggesting that it may be a modification in feline carnivores. The presentations of the digits is probably attributed to the need for pronation, supination, adduction and abduction which are important movements seen when hunting down prey. The appearance of the third phalanx together with some ligamental and muscular modifications enable retraction of its sharp pointed claws when not needed for hunting. Based on the previous studies on the tiger [20], leopard [17] and cat [15], it can be inferred from this study that the skeletal structures and arrangements of the African lion have common presentations with most feline species.

## Conclusion

This study on the Numerical and Morphological features of the African Lion’s (*Panthera leo leo*) pectoral limb skeleton presented numerical, detailed anatomical information and elucidated some similarities and differences when compared to the canine and feline species. While adding some valuable literature to science, it further serves as a baseline data for future scientific exposition on this species.

## Data Availability

The datasets used or analyzed during the current study are available from the corresponding author on reasonable request.
